# Dangerous Interference with the Ears

**Published:** 1876-10

**Authors:** 


### DANGEROUS INTERFERENCE WITH THE EARS.

Scarcely a day passes that does not bring us an ear trouble that is rendered difficult of cure, and sometimes dangerous to life even, that might have been avoided by a little forethought and care.

Teachers and parents often subject children to great danger of deafness by "boxing their ears." Applying the flattened hands to the ear with force, drives a column of air against the drum which ruptures it as quickly as is done in play with the green leaf that is laid between the hands and ruptured with a blow, to hear its loud report.

Precisely that which happens to the leaf, occurs to the ear-drum, causing a rent that is not only painful, but apt to result in lasting deafness. In a recent case, reported in the *Medical Journals*, death ensued from a disease of the ear caused by the teacher "boxing" the child's ear while at school. Rupture of the drum was quite frequent among artillerymen, in the early portion of the war, caused by the concussion produced by the heavy pieces of ordnance. Many cases of deafness were reported before the men learned to open their mouths widely while firing their pieces, so permitting the column of air to fall with equal force upon both surfaces of the drum. Pulling the ears of children is another bad habit which we find parents are prone to indulge in. The head should never be selected for corporal chastisement, —it contains too many sensative organs,—like the eye and ear—that are easily injured. There exist parts upon the person of a child, if he be reasonably chubby, that offer far greater facilities for a spank, and upon which the hand or slipper can be applied without danger of doing the evil doer any permanent personal injury.

Syringing the ear, for some fancied or real deposit of cerumen or wax, is often followed by sad consequences, particularly should no obstruction to the sensitive drum exist, in which event, a strong current of water is ruthlessly forced against that membrane, often inflicting either rupture or other serious injury. If occasion require the use of the syringe, the water should be thrown with the utmost care, and not directly into the opening, but rather against the side of the meatus or cavity, so that the force of the stream is broken ere it passes to the membrane. The effect of the syringing will be quite as good as though the stream had been thrown with greater force.

Picking wax from the ear with ear-spoon, pin, quill or other implement, is another exceedingly prevalent habit. Not infrequently the sensative drum is punctured or scratched with the instrument employed, or the hardened wax thrust against the membrane, to produce inflammatory disturbance. When the wax is not hard, it should never be removed at all. It is placed there for a purpose, and scooping it out, as many are fond of doing as a vague recreation, with the end of the spectacle frame, while interviewing a neighbor, is not the most innocent amusement that might be engaged in. In this manner, the lining membrane of the meatus is deprived of its proper lubricating material, when it soon becomes dry and inflamed, speedily drying up the wax, which increases the trouble, by means of its mechanical pressure, at last causing dullness in hearing, by parching the drum also.

Placing oils in the ear, for the purpose of softening the hardened wax, is another fruitful cause of deafness. The oil unites with the wax, attaches itself to the drum, coating it over with a sticky substance quite difficult of removal, when the oil soon becomes rancid, creating an irritation of the membrane. When it is desirable to soften a hardened secretion, use pure glycerine in the ear over night, confining it there with a pledget of cotton wool, and then carefully syringing it out with warm water next day, repeating the experiment as often as it may be necessary to remove the offending substance. Never poke at it with ear-spoon or other contrivance, remembering that rolling it around in the ear, against the sensitive lining membrane, or pushing it against the drum, does far more injury than the presence of the hardened substance would occasion.

Another means of frequent injury to the ears is found in the use of the nasal douche. When Thudichum introduced his douche, for washing out the nasal cavity, to the profession, he evidently did not foresee the evil consequences that were likely to arise from its indiscriminate use by the laity in general. Since the venders of quack nostrums for the cure (?) of catarrah, have adopted the instrument, sending a cheap, tin contrivance broadcast over the country, as a means of applying their so-called catarrh cures to the nose and throat of their numerously increasing victims, painful ear affections have become alarmingly numerous. If great care is not exercised in the use of this douche, the solution is forced into the middle-ear, causing buzzing in the ears, dulness of hearing, pain in the ear, and very frequently abscess of the ear, that subject the patient to intolerable pain, ruins his hearing, and endangers his life as well. The nasal douche is not a safe instrument in the hands of the patient, and should never be used save by the physician. It is an implement rarely or never employed by the physician even, by reason of its great danger of forcing fluids through the *eustachian tubes* into the middle-ear. Remember this, and discard the dangerous contrivance, before an accident befall you, such as we are daily seeing from its use.

Parents often make another very serious blunder in permitting their childrens' ears to discharge matter, under the impression that it is dangerous to arrest it, for fear it might break out elsewhere. Had the Supreme Being thought it best to attach a safety-valve of that character to our children, we are sure he would have selected a spot that would not endanger one of the most important of our senses. Discharges of matter from the ears, as a sequence of scarlet fever, measles or other diseased action, should *not* be permitted to continue, but the earliest moment should be seized to arrest its progress. Otorrhoea, as it is termed, when seated behind the drum—as occurs from scarlet fever and measles—soon carries away that membrane completely, causing deafness. When an aurist cannot be consulted, syringe the child's ear carefully, with soap and water, twice daily, not allowing a particle of the water to remain within the ear. Be careful to dry the ear thoroughly after every syringing. For this purpose, use a well worn linen napkin, introducing it within the ear over your little finger. A pledget of cotton that has been soaked in *tincture of iron*, and allowed to dry in the sun, may then be placed inside the ear and allowed to remain all day. In ordinary cases, taken in the beginning, this simple treatment will cure, if the child is otherwise in fair health. But at all events, have the difficulty arrested—for the continuance of the discharge will not only produce deafness, but will undermine the health of the child as well.

